# NaOH/Urea-Compatible Chitosan/Carboxymethylcellulose Films: Orthogonal Optimization of Packaging Properties

**DOI:** 10.3390/molecules30112279

**Published:** 2025-05-22

**Authors:** Chang Yu, Hui Sun, Lin Yao, Yunxuan Weng

**Affiliations:** 1 College of Light Industry Science and Engineering, Beijing Technology and Business University, Beijing 100048, China; 2230401018@st.btbu.edu.cn (C.Y.);; 2 Beijing Key Laboratory of Quality Evaluation Technology for Hygiene and Safety of Plastics, Beijing Technology and Business University, Beijing 100048, China

**Keywords:** NaOH/urea system, carboxymethyl cellulose, chitosan, orthogonal test, biodegradable film

## Abstract

Chitosan (CS)-based films have demonstrated significant potential as biodegradable packaging materials, but their suboptimal barrier and mechanical properties limit practical applications. In this study, CS/carboxymethyl cellulose (CMC) composite films were prepared using a NaOH/urea-based alkaline system. Optimal ratios (1.5% CS, 2% CMC, 2.5% NaOH, and 4% urea) were determined through an L_16_(4^4^) orthogonal array design. Fourier transform infrared spectroscopy (FTIR) and X-ray diffraction (XRD) analyses confirmed the formation of stable physical crosslinks between CS and CMC via hydrogen bonding. These interactions significantly enhanced mechanical properties (tensile strength: 46.08 MPa; elongation at break: 68%), improved thermal stability (maximum decomposition temperature: 304 °C), and superior barrier properties (water vapor transmission rate: 0.26 × 10^−5^ g/(m^2^·h·Pa); oxygen transmission rate: 1.12 × 10^−4^ g/(m^2^·s)). NaOH concentration exhibited the most pronounced influence on film performance. The composite film combines inherent biodegradability with excellent functional properties, offering a sustainable alternative to conventional petroleum-based packaging materials.

## 1. Introduction

Global plastic consumption currently stands at 430 million tons annually and is projected to reach 600 million tons by 2025, with non-biodegradable packaging materials dominating the market and causing severe environmental pollution [1]. This has driven research efforts toward biodegradable alternatives. Among these, chitosan (CS), the second most abundant natural polysaccharide, consists of β-(1→4)-linked 2-amino-2-deoxy-d-glucose units [2,3]. CS exhibits exceptional properties, including non-toxicity, biodegradability, biocompatibility, antimicrobial activity, and film-forming capability [4,5,6].

However, pure CS films suffer from limitations such as poor barrier performance, low mechanical strength, and inadequate thermal stability. They are soluble only in weakly acidic aqueous solutions [7]. In contrast, carboxymethyl cellulose (CMC), an anionic polysaccharide obtained through cellulose etherification [8], is non-toxic, cost-effective, and biodegradable, with high water solubility and excellent film-forming properties [9,10]. CMC films demonstrate high transparency, enhanced mechanical properties, and remarkable biocompatibility [11,12]. The complementary characteristics of CS and CMC suggest that their combination could overcome individual material limitations. Consequently, CS/CMC composite films fabricated by incorporating CMC into CS represent a promising eco-friendly packaging solution with wide-ranging applications.

Researchers have made significant progress in CS/CMC composite film research. Chen et al. [13] developed hydrochloric acid-dissolved CS/CMC films incorporated with blackberry anthocyanins and tea polyphenols, which showed enhanced tensile strength, improved elongation at break, and reduced UV–visible light transmittance compared to conventional films. Lan et al. [14] fabricated acetic-acid-based CMC/sodium alginate/CS films demonstrating 95.7% and 93.4% antimicrobial activity against Escherichia coli and Staphylococcus aureus, respectively.

Despite these advances, existing studies have primarily focused on acidic systems [14,15,16,17], while alkaline systems (e.g., NaOH/urea) remain relatively unexplored. Alkaline systems offer distinct advantages: they eliminate acidic residues’ environmental hazards and enhance intermolecular hydrogen bonding [15]. However, the current understanding is limited to single-material dissolution mechanisms, and the mechanism of intermolecular solubility of two-phase molecules and its regulation on the overall performance have not yet been clarified.

For instance, Hu et al. [16] found that NaOH/urea aqueous solutions effectively dissolve chitosan at −20 °C. In this system, urea disrupts intermolecular hydrogen bonds, preventing chain aggregation. Similarly, Cai et al. [17] demonstrated that alkaline solvents dissolve cellulose at low temperatures. Here, hydroxide ions weaken cellulose’s intermolecular hydrogen bonds, while urea disrupts the crystalline structure through water–cellulose interactions.

This study aims to develop CS/CMC composite films via a NaOH/urea system, systematically optimize their packaging-related properties through an orthogonal design, and elucidate the role of hydrogen bonding in enhancing performance.

## 2. Results and Discussion

### 2.1. Thickness and Apparent Analysis of (CS/CMC) Composite Film

The thickness of CS/CMC composite films ranged from 0.10 to 0.56 mm (Table 1). These variations are influenced by the concentrations of chitosan, carboxymethyl cellulose (CMC), urea, and sodium hydroxide, as well as the water content during film formation.

As summarized in Table 1, pure CS films exhibit a yellow color, whereas pure CMC films are white. The composite films retain transparency and homogeneity, with their color transitioning from colorless to light yellow as the CS content increases. This color shift may originate from the amino groups in chitosan, which absorb light in the visible spectrum.

### 2.2. FTIR Analysis of CS/CMC Composite Films

**Figure 1:**
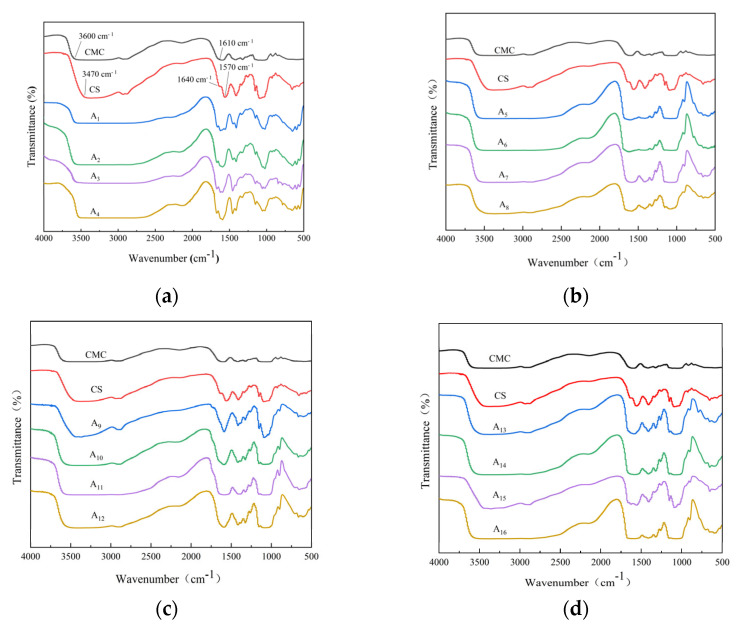
FTIR spectra of chitosan (CS), carboxymethyl cellulose (CMC), and CS/CMC composite films with varying CS additions (**a**) 1.0%, (**b**) 1.5%, (**c**) 2.0%, and (**d**) 2.5%.

As shown in Figure 1, the pure CS film exhibited characteristic peaks at 3470 cm^−1^, corresponding to NH and OH symmetric stretching vibrations as well as inter- and intramolecular hydrogen bonding [18]. The observed hydrogen bonding interactions were consistent with previous reports on chitosan-based materials [19]. Two distinct peaks at 1640 cm^−1^ and 1570 cm^−1^ were identified as amide I (C = O stretching vibration in the -NHCO- group) and amide II (NH bending vibration), respectively [18,19]. The presence of these amide signatures confirmed the existence of acetylated amine structures in the chitosan matrix.

In contrast, the pure CMC film exhibited a broader absorption band at 3600 cm^−1^, which originated from the stretching of O−H groups and inter- and intramolecular hydrogen bonding [19]. The absorption peak at 1610 cm^−1^ was attributed to the symmetric and asymmetric stretching vibrations of carboxyl groups (−COO^−^) [18,19].

No new characteristic bands were generated in the FTIR spectra of the composite membranes, suggesting a physical interaction between CS and CMC. Under alkaline conditions, NaOH decomposed into Na⁺ and OH-, which neutralized -NH_3_⁺ in CS as well as −COO^−^ in CMC, changing the charged state of the molecules and affecting the intermolecular interactions [20]. Meanwhile, urea forms hydrogen bonds with CS and CMC and water, which reduces phase separation and promotes uniform dispersion and interaction of CS and CMC in the system [21]. In the CS/CMC films, the intermolecular hydrogen bonding between CMC and CS resulted in peaks of the O-H and N-H stretching bands between 3470 and 3640 cm^−1^. The amide I band at 1640 cm^−1^ was red-shifted, and the FTIR peaks of CS (−NH at 3470 cm^−1^) and CMC (−COO^−^ at 1610 cm^−1^) were shifted by 5–50 cm^−1^, indicating that intermolecular hydrogen bonding was formed between the amino group in CS and carboxylic acid group in CMC, which produced a strong physical hydrogen bonding interactions. This indicated that the CS/CMC composite film was successfully synthesized. FTIR analysis was critical to confirm the absence of chemical crosslinking and identify hydrogen bonding interactions between CS and CMC, which directly correlate with mechanical and barrier properties. Furthermore, CS and CMC formed a stabilizing network structure through hydrogen bonding, which has been verified in studies [20,22,23], and provided a structural basis for the excellent mechanical properties, thermal stability, and barrier properties of the composite film. Similar results have been reported in other studies. For example, Wu et al. [24] found that the hydrogen bonding interactions between CS and CMC significantly improved the mechanical properties of the composite membrane. Physical crosslinking via hydrogen bonding was preferred over chemical methods to retain the biocompatibility and biodegradability of CS/CMC, avoiding potential toxicity from crosslinking agents.

### 2.3. XRD Analysis of CS/CMC Composite Films

**Figure 2:**
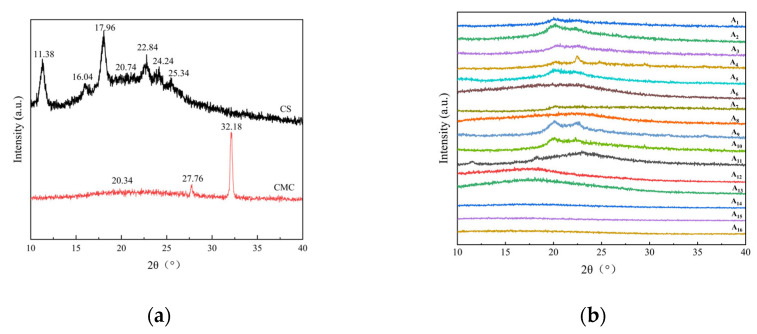
X-ray diffraction (XRD) patterns of (**a**) pure CS and CMC films, and (**b**) CS/CMC composite films with varying NaOH concentrations.

As shown in Figure 2a, the pure CS films exhibited distinct crystallization peaks at 2θ = 11.38°, 16.04°, and 20.74°, which were associated with the ordered arrangement of CS molecular chains and intramolecular hydrogen bonding. The pure CMC film showed broad diffraction peaks near 2θ = 20.66°, indicating its amorphous nature.

In the CS/CMC composite films (Figure 2b), the characteristic peaks were shifted in position, and the crystallinity was reduced by 22–81% compared to the pure films. The addition of CMC disrupted the orderly arrangement of CS molecular chains, resulting in broader and less intense crystalline peaks. This was attributed to carboxymethyl groups on CMC chains hindering hydrogen bond formation between CS molecules.

The NaOH/urea solvent system was found to significantly influence the crystalline structure. Variations in alkali concentration altered the hydrogen bond network, with excessive NaOH leading to more disordered structures. These observations confirmed that the composite films’ crystallinity was strongly dependent on both composition and processing conditions [25].

### 2.4. Thermogravimetric Analysis of CS/CMC Composite Films

The thermal stability of the films was evaluated using a thermogravimetric analyzer (STA7200, Hitachi) under a nitrogen atmosphere (flow rate of 20 mL/min). The temperature was increased from 40 to 800 °C at a heating rate of 10 °C/min.

**Figure 3:**
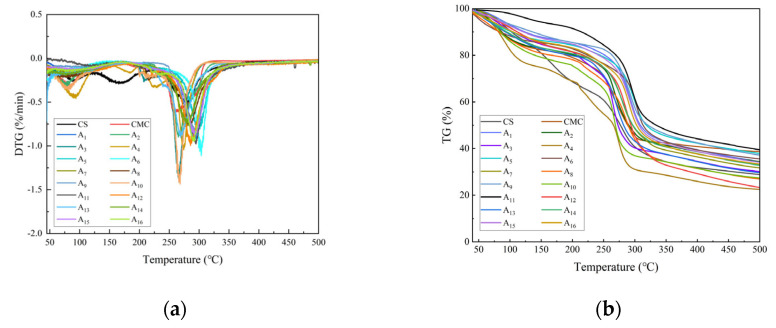
Thermogravimetric analysis of CS, CMC, and CS/CMC composite films: (**a**) DTG curves, (**b**) TG curves. Tests were conducted under nitrogen atmosphere at a heating rate of 10 °C/min.

As shown in Figure 3a,b, the thermal decomposition of composite films occurred in four stages. The first stage (50−150 °C) corresponded primarily to volatile loss and water evaporation. The second stage (150–220 °C) involved hydrogen bond breakage between CMC and CS, accompanied by urea decomposition (NH_3_ and CO_2_ release). The third stage (220−400 °C) is associated with rapid weight loss due to the decomposition of CMC glucose units and CS glucosamine units. The decomposition onset temperature of composite films (A_4_, A_5_, A_7_, etc.) ranged from 245 to 262 °C, 12–18% higher than pure CS (220 °C) and CMC (230 °C). The fourth stage (>400 °C) represents the slow weight loss from residual carbides and ash.

Composite films demonstrated significantly higher decomposition temperatures and enhanced thermal stability compared to pure CS and CMC films. This improvement was attributed to dense hydrogen-bonded networks formed by optimal CS/CMC ratios in the NaOH/urea system, which restricted molecular chain mobility.

### 2.5. DSC Analysis of CS/CMC Composite Film

**Figure 4:**
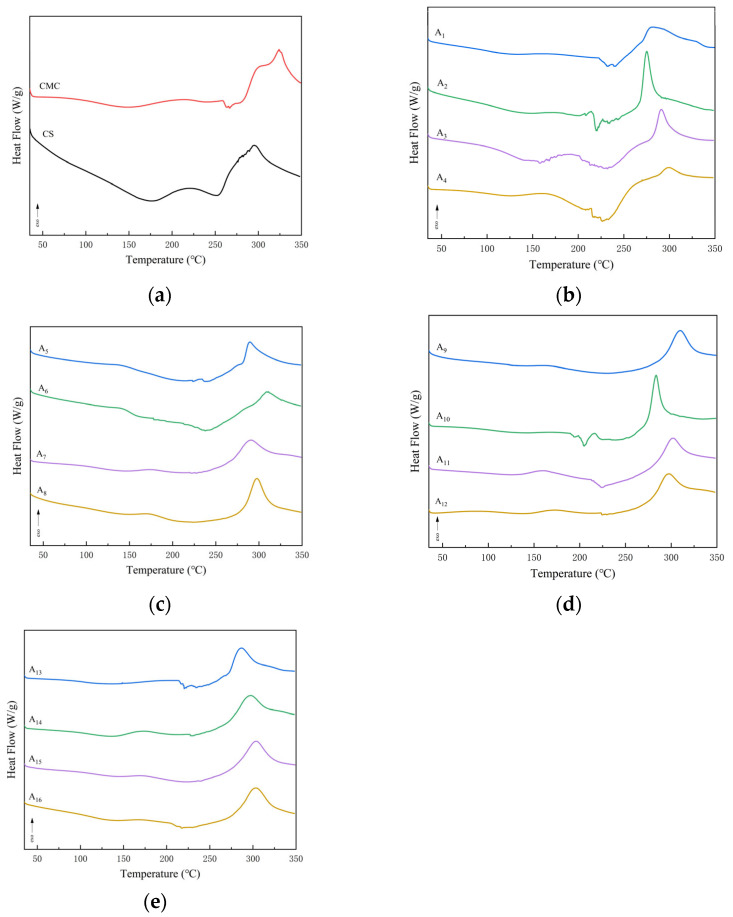
DSC of CS film, CMC film, and CS/CMC composite film with different addition amounts: (**a**) pure CS and CMC films; (**b**) composite film with 1.0% CS addition; (**c**) 1.5% CS addition; (**d**) 2.0% CS addition; (**e**) 2.5% CS addition.

As shown in Figure 4a, the pure CS membrane showed two heat absorption peaks at 177 °C and 252 °C, which corresponded to the conformational transition of the molecular chain and the deacetylation process, respectively [26], and the exothermic peak at 295 °C was attributed to the thermal decomposition reaction of the CS backbone, and this result was in agreement with the results of thermogravimetric analysis. The pure CMC membrane has two heat absorption peaks at 149 °C and 264 °C, as well as an exothermic peak at 324 °C. The cellulose membrane has been characterized by a series of thermal decomposition reactions. The thermal decomposition of cellulose consists of a series of reactions including dehydration, decarboxylation, decarbonylation, and a complex pyrolysis process of glycosidic, C-H, and C-O-C bond breaking [26,27].

As can be seen from Figure 4b–e, the first heat absorption peak of the composite films appeared at 134–160 °C in comparison to the pure CS films, which shifted toward lower temperatures. This phenomenon suggests that the NaOH/urea system breaks some of the intermolecular hydrogen bonds and reduces the initial thermal stability of the molecular chains [28]. The second heat absorption peak temperature was in the range of 194–238 °C, which was lower than that of the pure CS film, and this change reflected the interaction between CS and CMC, and also confirmed that the incorporation of CMC disrupted the crystalline structure of CS. The exothermic peak temperatures of the composite membranes were in the range of 275–311 °C, fluctuating around the exothermic peak temperature of 295 °C for the pure CS membrane. This indicates that the thermal decomposition behavior of the composite membrane is affected by a combination of factors, including the interaction between CS and CMC, the change in crystallinity, the role of the solvent system, etc., and these factors work together to make the thermal decomposition behavior of the composite membrane change significantly.

In conclusion, the DSC analysis results confirmed the formation of a new composite structure between CS and CMC molecules through the NaOH/urea system. The thermal decomposition behavior of this composite structure retained the characteristics of each component and produced significant synergistic effects. In addition, the results are corroborated by the formation of hydrogen bonds detected by FTIR and the decrease in crystallinity shown by XRD, which provide an important theoretical basis for the synergistic optimization of the mechanical properties, barrier properties, and thermal stability of the composite films.

### 2.6. Mechanical Properties of CS/CMC Composite Films

**Figure 5:**
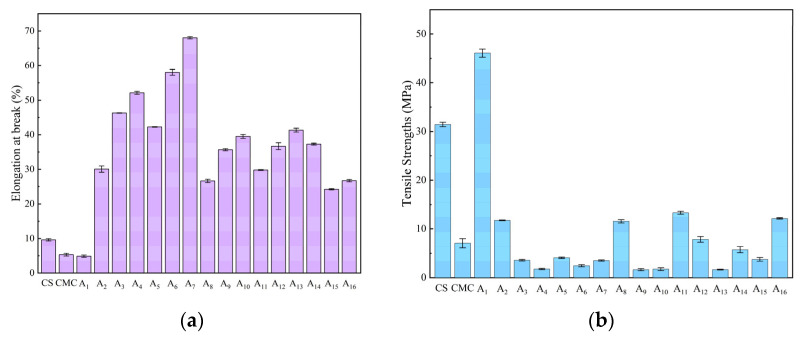
Mechanical properties of CS, CMC, and CS/CMC composite films: (**a**) elongation at break (%), (**b**) tensile strength (MPa).

The mechanical performance was evaluated through tensile strength and elongation at break measurements (Figure 5). Pure CS film exhibited 31.42 MPa tensile strength and 9.61% elongation, while pure CMC film showed 7.06 MPa and 5.32%. NaOH/urea-prepared composites demonstrated superior properties, with A_7_ reaching 64.86% elongation and A_1_ achieving 46.08 MPa tensile strength. This enhancement was attributed to optimized CS/CMC mixing and hydrogen-bonded network formation.

However, maximum tensile strength (46.08 MPa) was achieved only at 1% concentrations of both CS and CMC. Higher CMC concentrations reduced tensile strength significantly (e.g., 11.77 MPa at 1.5% CMC) compared to pure CS films (31.42 MPa). This behavior reflects their distinct roles: CS provides mechanical support through hydrogen bonding sites, while CMC primarily bears external forces. Notably, low concentrations of CMC, NaOH, and urea increased tensile strength, whereas higher concentrations enhanced flexibility (up to 68% elongation) and reduced brittleness. XRD analysis confirmed that reduced crystallinity in composite films led to looser molecular chain arrangement, decreasing rigidity while improving stretchability [29]. This is consistent with the findings of He et al. [30] on cellulose/chitosan composite films prepared in LiOH/urea aqueous solution.

The mechanical properties of CS/CMC composite films prepared under alkaline conditions (A_1_: tensile strength 46.08 MPa, elongation at break 4.88%; A_7_: tensile strength 7.84 MPa, elongation at break 68.00%) were superior to those of Valizadeh et al. [31] prepared in a conventional acetic acid system and close to those of low-density polyethylene (LDPE, tensile strength 8–20 MPa, elongation at break 100–600%). Its high tensile strength is suitable for primary food packaging (e.g., dry product bags), while its high ductility can be applied to flexible packaging (e.g., cling film).

### 2.7. Moisture Content, Water Absorption, and Solubility of CS/CMC Composite Films

**Figure 6:**
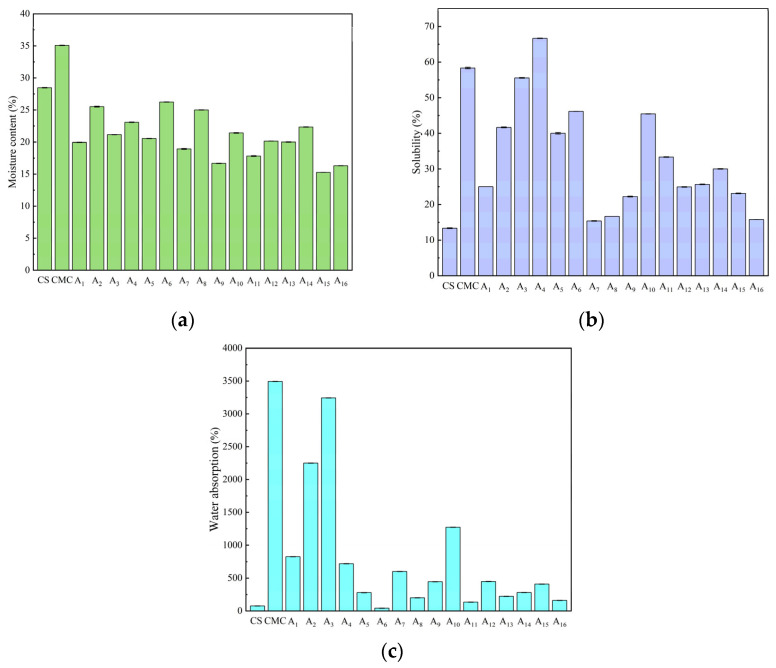
(**a**) Moisture content, (**b**) solubility, and (**c**) water absorption of CS, CMC, and CS/CMC composite films.

Water content critically influences film flexibility and water resistance in humid conditions. Pure CS films exhibited high water content (Figure 6a) due to strong hydrogen bonding between water molecules and CS functional groups (hydroxyl and amino groups). Composite films showed reduced water content (12.44–31.02%), with A_2_ and A_9_ representing the highest and lowest values, respectively. This reduction resulted from optimized CS/CMC hydrogen bonding that limited water absorption.

Film solubility, closely related to water resistance, showed complex concentration dependence (Figure 6b). The A_4_ composite film reached maximum solubility (15.38%), reflecting CMC’s inherent hydrophilicity (pure CMC: 58.33%), while A_7_ showed minimum solubility (62% reduction vs. pure CS) due to its compact structure. Composite films exhibited significantly greater water absorption than pure CS (82–4322× increase, Figure 6c), attributable to the hydrophilic nature of both polymers [32].

Both composition and processing conditions governed water sensitivity. NaOH concentration affected intermolecular cross-linking via pH modification, while urea modulated hydrogen bond stability. Similar results were reported in a study by Liu et al. [20]. Optimal concentrations promoted dense networks that reduced water uptake, whereas excess amounts disrupted these networks, increasing hydrophilicity. These interdependent factors collectively determine the films’ water-related performance for packaging applications.

### 2.8. Water Vapor Permeability (WVP) and Oxygen Transmission Rate (OTR) of CS/CMC Composite Films

**Figure 7:**
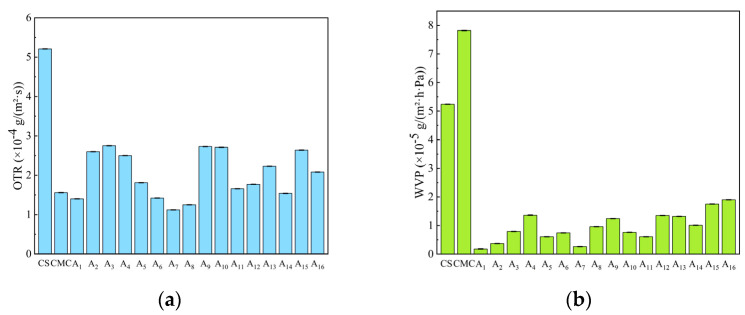
(**a**) Water vapor transmission rate, and (**b**) oxygen transmission rate of CS, CMC, and CS/CMC composite films.

Oxygen permeability (OTR) is an important indicator for evaluating the freshness retention ability of composite films. As shown in Figure 7a, the pure CS film exhibited an OP of 5.21 × 10^−4^ g/(m^2^·s), and the incorporation of CMC into CS significantly reduced the OP of CS/CMC composite films.

Among these, the CS composite film with 1.5% CMC addition demonstrated excellent oxygen barrier performance. The OTR of composite films initially decreased but then increased with further CMC addition, with the A_7_ composite film showing the lowest OP and best barrier performance.

This is attributed to the formation of a denser network structure in optimally proportioned NaOH/urea solutions, which reduces oxygen transmission. However, excessive CMC addition disrupts the CS crystalline structure and increases film hydrophilicity, thereby raising oxygen transmission rates.

Effective oxygen barrier performance delays package content oxidation, maintains product quality and freshness, and enhances the value of composite films for food packaging applications.

Water vapor permeability (WVP) is a key parameter for assessing composite film barrier performance, directly influencing packaging material moisture resistance. Figure 7b shows that while pure CS and CMC films exhibit higher WVP values, all CS/CMC composite films demonstrate lower WVP values than single-component films, indicating superior water vapor barrier performance.

This occurs because the inherent hydrophilic groups in CS and CMC create relatively weak water vapor barriers. However, when composited in the NaOH/urea system, the physical cross-linking between the polymer chains produces a denser dry film surface that hinders water vapor transport, thereby reducing the WVP value.

Effective water vapor barrier performance prevents moisture-induced deterioration of packaged contents, prolongs product shelf life, and represents a key advantage for composite film packaging application

### 2.9. UV-Blocking Properties

UV-blocking performance is an important index for measuring how well packaging materials protect contents from UV radiation damage. Analysis of transmittance at 280 nm and 320 nm (*T*_280_ and *T*_320_) showed CS/CMC composite films had significantly better UV-shielding performance than pure CS or CMC films.

As shown in Table 2, the composite films A_4_, A_9_, A_12_, A_13_, A_14_, and A_15_ had lower 280 nm transmittance than single-component films.

The 280 nm and 320 nm values of A_12_ and A_15_ films approached 0%, indicating nearly complete UV blockage. This performance is attributed to hydrogen and amide bonding networks between CS and CMC that effectively absorb and scatter UV radiation [33,34], thereby enhancing UV-blocking performance.

Furthermore, NaOH and urea addition optimized film structure by promoting CS-CMC cross-linking, forming denser networks that further enhanced UV barrier performance. These findings agree with the literature reports. For example, Wu et al. [24] demonstrated that CS-CMC hydrogen bonding significantly enhances composite film UV barrier properties. Yang et al. [11] also reported that optimal CS/CMC ratios form dense networks that improve UV-blocking properties.

Compared to the *T*_280_ (>10%) of CS/CMC films prepared in hydrochloric acid solution [13], alkaline-prepared composite films showed significantly lower *T*_280_ values.

### 2.10. Statistical Analysis of Orthogonal Experiment

In this study, mechanical properties, UV-blocking performance, WVP, and OP were evaluated, as shown in Table 2. The orthogonal test results (Table 3 and Table 4) were analyzed using range analysis and variance analysis.

Data from previous tests were synthesized to determine the optimal composite film formulation. The range value (*R*) reflects factor importance in the experiment. Larger R values indicate greater factor influence on the measured parameters, typically identifying main factors. Conversely, smaller R values suggest a lesser influence on the response variables.

Analysis of variance (ANOVA) was employed to assess the statistical significance of each factor’s effect on the response variables. Typically, *p*-values > 0.05 indicate non-significant differences, *p*-values < 0.05 denote significant factor effects, and *p*-values < 0.01 represent highly significant effects on the response variables.

ANOVA results demonstrated that NaOH concentration (Factor C, *R* = 16.34) most significantly affected tensile strength by directly modulating intermolecular hydrogen bond density, while urea (Factor D) improved flexibility through crystalline structure disruption (Table 3). Furthermore, ANOVA revealed significant NaOH effects on both WVP (*p* = 0.008) and OP (*p* = 0.025) (Table 4), confirming the alkaline system’s crucial role in barrier properties.

By combining the test results in Table 2 with the range analysis in Table 3 and the variance analysis in Table 4 for each factor, we obtained the following results.

For Factor A, which significantly affects both water vapor transmission rate and oxygen transmission rate, A2 was selected. Although Factor A showed no significant effect on tensile strength, elongation at break, *T*_280_, and *T*_320_, A2 was still chosen based on the range analysis results. In summary, to ensure good overall performance of the composite film, the chitosan concentration should be set at the A2 level.

Factor B significantly affects the water vapor transmission rate, with B2 being optimal. For tensile strength, elongation at break, *T*_280_, *T*_320_, and oxygen transmission rate, where Factor B shows no significant effect, B3 was selected based on range analysis. The evaluation shows that B2 provides an optimal water vapor transmission rate and better UV-barrier properties and oxygen transmission rate, but poorer mechanical properties. B3 offers better balanced mechanical properties including tensile strength and elongation at break, moderately good UV barrier properties, and superior water vapor and oxygen transmission rates. Therefore, B3 was selected as the optimal carboxymethyl cellulose concentration to ensure good overall film performance.

Factor C showed no significant effects on the composite film. Based on range analysis of mechanical properties, UV barrier properties, water vapor, and oxygen transmission rates, both C1 and C4 were considered, with C4 being ultimately selected. Thus, the sodium hydroxide concentration was set at the C4 level to ensure good overall film performance.

Factor D most significantly affected the oxygen transmission rate as the primary factor, leading to the selection of D1. It also significantly influenced the water vapor transmission rate as a secondary factor. For tensile strength, elongation at break, *T*_280_, and *T*_320_, where no significant effects were observed, D1 was still selected based on range analysis. Considering mechanical properties, UV barrier properties, water vapor, and oxygen transmission rates as evaluation criteria, and to ensure good overall performance, the urea concentration was set at the D1 level.

Based on the above analysis, the optimal composite film formulation is A2B3C4D1, consisting of 1.5% chitosan, 2% carboxymethyl cellulose, 2.5% sodium hydroxide, and 4% urea. This formulation demonstrates better mechanical and barrier properties, with moderate additive concentrations that balance all performance indicators.

## 3. Materials and Methods

### 3.1. Materials

Chitosan (degree of deacetylation ≥ 95, determined by elemental analysis according to ASTM F210318 [35]; viscosity: 100–200 mPa·s), glacial acetic acid, and sodium hydroxide were obtained from Shandong Keyuan Biochemical Co., Ltd. (Heze, China). Sodium carboxymethyl cellulose (viscosity: 800–1200 mPa·s) was obtained from Shanghai Yuanye Bio-Technology Co., Ltd. (Shanghai, China). Urea and glycerol were supplied by Fuchen Chemical Reagent Co., (Tianjin, China).

### 3.2. Film Preparation

Pure CS films were prepared by dissolving CS (1.0–2.5 wt%) in 1 vol% acetic acid under stirring at 25 °C for 2 h. Similarly, pure CMC films were obtained by dissolving carboxymethyl cellulose (1.0–2.5 wt%) in deionized water. Glycerol (30 wt% of dry polymer mass) was added to both solutions, followed by 30 min stirring and defoaming. The solutions were cast onto Petri dishes (13 × 13 cm) and dried at 40.0 ± 0.5 °C.

For composite films, CS and CMC solutions were combined at a 1:1 volume ratio. After adding glycerol (30 wt%), the mixture was stirred for 30 min and defoamed (vacuum, 30 min). The resulting solution was cast onto Petri dishes and dried at 40.0 ± 0.5 °C. The dried films were neutralized with 1 M acetic acid or NaOH, rinsed with deionized water, and air-dried. The percentages of each component in the dry film are shown in Table 5.

Control films were prepared in 1 vol% acetic acid (pH 2.5 ± 0.1) following the same procedure for comparison.

### 3.3. Orthogonal Experimental Design

The comprehensive performance of CS/CMC composite films was evaluated through an L_16_(4^4^) orthogonal array design. Key evaluation metrics were selected to represent critical packaging material characteristics, including mechanical properties (tensile strength and elongation at break), water vapor transmission rate (WVP), oxygen transmission rate (OTR), and UV-blocking performance (transmittance at 280 nm and 320 nm). These parameters were chosen to systematically assess the film’s mechanical strength, flexibility, moisture resistance, freshness preservation capability, and UV protection efficiency.

#### 3.3.1. Factors and Levels

Four key factors were identified for optimization: CS concentration (Factor A: 1.0–2.5% *w*/*v*), CMC concentration (Factor B: 1.0–2.5% *w*/*v*), NaOH concentration (Factor C: 2.0–8.0% *w*/*v*), and urea concentration (Factor D: 4.0–10.0% *w*/*v*). Each factor was assigned four equally spaced levels, with the complete level design presented in Table 6. The L_16_(4^4^) orthogonal array was adopted to efficiently explore this four-factor, four-level design space while maintaining statistical rigor.

#### 3.3.2. Experimental Matrix

A 16-run experimental design was constructed using the L_16_(4^4^) orthogonal array. (Table 7), with each experimental group replicated three times to ensure statistical reliability.

### 3.4. Characterization and Testing

#### 3.4.1. Thickness and Appearance of CS/CMC Composite Film

Five points were randomly selected on the composite film with a thickness gauge, and the average value was taken as the thickness of the film, in mm. The appearance of the composite film was photographed by a digital camera.

#### 3.4.2. FTIR Analysis

FTIR spectra were recorded using a Nicolet iS10 spectrometer (Thermo Fisher Scientific, Waltham, MA, USA). The films were analyzed in the spectral range of 500–4000 cm^−1^ with a resolution of 4 cm^−1^. Each spectrum was acquired by averaging 32 consecutive scans to ensure signal-to-noise ratio.

#### 3.4.3. X-Ray Diffraction (XRD)

Crystalline structures were characterized by XRD (Rigaku SmartLab, Tokyo, Japan) with Cu Kα radiation (λ = 1.5406 Å) at 40 kV and 30 mA. Scans were performed in the 2θ range of 10–40° with a scanning speed of 5°/min. The samples were mounted on a zero-background silicon holder to minimize substrate interference. The formula for calculating the degree of crystallinity is as follows:

(1) $$Crystallinity\;(\%)=\left(\frac{A_{crys}}{A_{total}}\right)\times 100\%$$

where *A_crys_* is the XRD integrated area of the crystalline portion; *A_total_* is the total XRD integrated area.

#### 3.4.4. Thermogravimetric Analysis

The thermal stability of the films was evaluated using a thermogravimetric analyzer (STA7200, Hitachi, Tokyo, Japan). The temperature was increased from 40 to 500 °C at a heating rate of 10 °C/min under a nitrogen atmosphere with a flow rate of 20 mL/min.

#### 3.4.5. Differential Scanning Calorimetry (DSC)

The composite films were analyzed by differential scanning calorimeter (Q100, TA). The test conditions were nitrogen atmosphere, temperature range 40–350 °C, heating rate 10 °C/min.

#### 3.4.6. Mechanical Properties

Tensile properties were measured according to GB/T 13022-91 [36] using a universal testing machine (CMT6104, MTS Systems, Shenzhen, China). Film specimens (10 × 130 mm) were clamped with a 50 mm gauge length and tested at a crosshead speed of 5 mm/min. Five replicates were measured for each sample group, and the average values of tensile strength and elongation at break were calculated. Film thickness was determined using a digital micrometer.

#### 3.4.7. Moisture Content, Water Absorption and Solubility

The water content of the films was determined using the mass loss method [37]. Film samples (2 cm × 2 cm) were weighed (*W*_1_) and dried at 105 °C until constant weight (*W*_2_), and the moisture content was calculated using Equation (2). For solubility testing, dried films were immersed in distilled water for 24 ± 1 h and removed, and surface moisture was blotted before weighing (*W*_3_). The films were then redried to constant weight (*W*_4_), and solubility was calculated using Equations (3) and (4). All measurements were recorded with an accuracy of ±0.01 g.

(2) $$Moisture\;content(\%)=\frac{W_{1}-W_{2}}{W_{1}}\times 100\%$$

(3) $$Water\;absorption(\%)=\frac{W_{3}-W_{2}}{W_{2}}\times 100\%$$

(4) $$Solubility(\%)=\frac{W_{2}-W_{4}}{W_{2}}\times 100\%$$

#### 3.4.8. Water Vapor Transmission Rate (WVP)

The water vapor transmission rate was measured according to GB/T 1037-2021 [38], with modifications based on the methodology of Manjushree et al. [39]. Glass vials (10 mL) were filled with anhydrous calcium chloride (dried) and sealed with the test films, maintaining a 3 mm gap between the desiccant and film surface. The assemblies were placed in desiccators containing saturated aqueous solutions at 25 °C. Weight measurements were taken at 24 h intervals (accuracy: ±0.01 g). The WVP was calculated from the linear regression of mass gain versus time using Equation (5):

(5) $$WVP=\frac{K\times D}{S\times ∆P}$$

where *K* is the slope of the obtained straight line (g/h); *D* is the thickness of the composite Film (mm); *S* is the area of the bottle mouth (m^2^); Δ*P* is the vapor pressure on both sides of the composite film at 25 °C (Pa).

#### 3.4.9. Oxygen Permeability (OTR)

The oxygen permeability (OTR) was determined based on iron oxidation, following the deoxidizer adsorption method described by Zhang et al. [40]. A mixture of sodium chloride (1.5 g), activated carbon (1 g), and reduced iron powder (0.5 g) was loaded into brown glass vials (neck diameter: 2 cm). Film samples were heat-sealed over the vial openings, and the assemblies were stored at 25 °C and 90% relative humidity (RH) for 48 h. Triplicate tests were conducted for each sample, and the OTR was calculated using Equation (6).

(6) $$OTR=\frac{m-m_{0}}{t\times s}$$

#### 3.4.10. Ultraviolet–Visible Spectroscopy

UV–Vis transmittance spectra of the films were recorded at 25 °C using a UV–Vis spectrophotometer. Film samples were cut into strips (1 cm × 5 cm). Background correction was performed using an empty cuvette as a reference. Each film strip was mounted on a cuvette, and transmittance was measured from 200 to 800 nm.

## 4. Conclusions

In this study, CS/CMC composite films prepared in the NaOH/urea system achieved optimal performance at 1.5% CS, 2% CMC, 2.5% NaOH, and 4% urea. The alkaline system avoided acidic solvent residues while enhancing hydrogen bonding, yielding films with exceptional mechanical properties (46.08 MPa tensile strength, 68% elongation), barrier performance (WVP: 0.26 × 10^−5^ g/(m^2^·h·Pa); OTR: 1.12 × 10^−4^ g/(m^2^·s)), and thermal stability (304 °C decomposition temperature). These results position the film as a sustainable alternative to petroleum-based packaging. Future research will systematically evaluate the biodegradability and practical application potential of CS/CMC composite materials by verifying the hydrophilicity of the films, conducting composting degradation experiments, and testing their food preservation effects under real storage conditions.

## Figures and Tables

**Table 1:** Thickness (mean ± SD, mm) and visual appearance of CS, CMC, and CS/CMC composite films.

Run No.	Thickness (mm)	Apparent
CS	0.11 ± 0.01	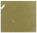
CMC	0.11 ± 0.01	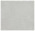
A_1_	0.10 ± 0.01	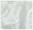
A_2_	0.15 ± 0.01	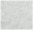
A_3_	0.19 ± 0.02	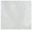
A_4_	0.22 ± 0.01	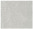
A_5_	0.16 ± 0.02	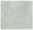
A_6_	0.24 ± 0.02	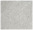
A_7_	0.30 ± 0.02	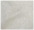
A_8_	0.36 ± 0.03	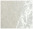
A_9_	0.38 ± 0.03	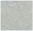
A_10_	0.32 ± 0.02	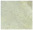
A_11_	0.27 ± 0.02	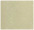
A_12_	0.40 ± 0.03	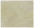
A_13_	0.33 ± 0.03	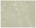
A_14_	0.38 ± 0.01	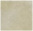
A_15_	0.43 ± 0.02	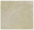
A_16_	0.46 ± 0.02	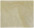

**Table 2:** Orthogonal test results for mechanical, barrier, and UV-blocking properties of CS, CMC, and CS/CMC composite films.

Run No.	Experimental Design				Response Variables					
	A: CS % (*w*/*v*)	B: CMC % (*w*/*v*)	C: NaOH % (*w*/*v*)	D: Urea % (*w*/*v*)	Tensile Strength (MPa)	Elongation at Break (%)	*T*_280_ (%)	*T*_320_ (%)	WVP (×10^−5^ g/(m^2^·h·Pa))	OTR (×10^−4^ g/(m^2^·s))
A_1_	1 (1.0%)	1 (1.0%)	1 (1.0%)	1 (4.0%)	46.08	4.88	2.44	3.80	0.18	1.40
A_2_	1	2 (1.5%)	2 (1.5%)	2 (8.0%)	11.77	30.06	14.48	19.19	0.37	2.60
A_3_	1	3 (2.0%)	3 (2.0%)	3 (12.0%)	3.54	46.27	4.07	7.21	0.79	2.75
A_4_	1	4 (2.5%)	4 (2.5%)	4 (16.0%)	1.76	52.11	0.73	1.29	1.36	2.50
A_5_	2 (1.5%)	1	2	3	4.06	42.22	4.30	5.73	0.61	1.81
A_6_	2	2	1	4	2.45	58.04	6.67	8.40	0.74	1.42
A_7_	2	3	4	1	3.47	68.00	1.34	2.14	0.26	1.12
A_8_	2	4	3	2	11.57	26.64	4.72	6.72	0.96	1.25
A_9_	3 (2.0%)	1	3	4	1.62	35.67	0.77	1.43	1.24	2.73
A_10_	3	2	4	3	1.74	39.51	2.54	4.87	0.76	2.71
A_11_	3	3	1	2	13.3	29.77	4.36	6.50	0.61	1.66
A_12_	3	4	2	1	7.84	36.68	0.36	0.61	1.35	1.77
A_13_	4 (2.5%)	1	4	2	1.64	41.34	0.83	1.33	1.32	2.23
A_14_	4	2	3	1	5.73	37.30	0.62	1.08	1.01	1.54
A_15_	4	3	2	4	3.72	24.25	0.18	0.40	1.75	2.64
A_16_	4	4	1	3	12.14	26.73	1.27	2.35	1.90	2.08

**Table 3:** Range analysis of orthogonal test results.

Response Variable		Experimental Factors			
		A: CS	B: CMC	C: NaOH	D: Urea
Tensile strength (MPa)	*k*_1_	15.79	13.35	18.49	15.78
	*k*_2_	5.39	5.42	6.85	9.57
	*k*_3_	6.13	6.01	5.62	5.37
	*k*_4_	5.81	8.33	2.15	2.39
	*R*	10.40	7.93	16.34	13.39
	Order of priority	C > D > A > B			
	Optimization	A1	B1	C1	D1
Elongation at break (%)	*k*_1_	33.33	31.03	29.86	36.71
	*k*_2_	48.72	41.23	33.30	31.95
	*k*_3_	35.41	42.07	36.47	38.68
	*k*_4_	32.41	35.54	50.24	42.52
	*R*	16.32	11.04	20.38	10.57
	Order of priority	C > A > B > D			
	Optimization	A2	B3	C4	D4
*T*_280_ (%)	*k*_1_	5.43	2.09	3.69	1.09
	*k*_2_	4.16	6.08	4.83	6.10
	*k*_3_	2.01	2.39	2.55	3.05
	*k*_4_	0.73	1.77	1.26	2.09
	*R*	4.71	4.31	3.57	5.01
	Order of priority	D > A > B > C			
	Optimization	A4	B4	C4	D1
*T*_320_ (%)	*k*_1_	7.87	3.07	5.26	5.26
	*k*_2_	5.60	8.39	6.48	8.44
	*k*_3_	3.35	3.91	4.11	5.04
	*k*_4_	1.29	2.74	2.26	2.88
	*R*	6.58	5.64	4.23	5.56
	Order of priority	A > B > D > C			
	Optimization	A4	B4	C4	D4
WVP (×10^−5^ g/(m^2^·h·Pa))	*k*_1_	0.68	0.84	0.86	0.70
	*k*_2_	0.64	0.72	1.02	0.82
	*k*_3_	0.99	0.85	1.00	1.02
	*k*_4_	1.50	1.39	0.93	1.27
	*R*	0.85	0.67	0.16	0.57
	Order of priority	A > B > D > C			
	Optimization	A2	B2	C1	D1
OTR (×10^−4^ g/(m^2^·s))	*k*_1_	2.31	2.04	1.64	1.46
	*k*_2_	1.40	2.07	2.20	1.93
	*k*_3_	2.22	2.04	2.07	2.34
	*k*_4_	2.12	1.90	2.14	2.32
	*R*	0.91	0.17	0.56	0.88
	Order of priority	A > D > C > B			
	Optimization	A2	B4	C1	D1

**Table 4:** Analysis of variance (ANOVA) for mechanical and barrier properties in the orthogonal experiment.

Source of Variance	Implicit Variable	Sum of Squared Deviations	Degree of Freedom	Mean Square	*F*	*p* *
A: CS	Tensile strength	301.945	3	100.648	0.932	0.522
	Elongation at break	694.891	3	231.63	0.650	0.634
	*T*_280_	54.411	3	18.137	5.171	0.105
	*T*_320_	98.135	3	32.712	5.866	0.090
	WVP	1.875	3	0.625	32.975	0.008
	OTR	2.077	3	0.692	15.418	0.025
B: CMC	Tensile strength	156.147	3	52.049	0.482	0.718
	Elongation at break	322.13	3	107.377	0.301	0.825
	*T*_280_	48.159	3	16.053	4.576	0.122
	*T*_320_	81.576	3	27.192	4.876	0.113
	WVP	1.083	3	0.361	19.052	0.019
	OTR	0.070	3	0.023	0.519	0.698
C: NaOH	Tensile strength	603.983	3	201.328	1.864	0.311
	Elongation at break	957.717	3	319.239	0.895	0.535
	*T*_280_	26.683	3	8.894	2.536	0.232
	*T*_320_	36.101	3	12.034	2.158	0.272
	WVP	0.066	3	0.022	1.166	0.451
	OP	0.780	3	0.260	5.793	0.092
D: Urea	Tensile strength	404.415	3	134.805	1.248	0.430
	Elongation at break	231.841	3	77.28	0.217	0.879
	*T*_280_	54.645	3	18.215	5.193	0.105
	*T*_320_	100.416	3	33.472	6.002	0.088
	WVP1	0.756	3	0.252	13.291	0.031
	OTR	2.063	3	0.688	15.314	0.025

* Note: *p* > 0.05, not significant; *p* < 0.05, significant; *p* < 0.01, highly significant.

**Table 5:** Percentage of each component in dry film.

Compose	A: CS	B: CMC	C: NaOH	D: Urea
Dry film mass percentage (*w*/*w*%)	4.3–22.3	4.8–22.6	4.8–22.6	4.3–20.9

**Table 6:** L_16_(4^4^) orthogonal array design for CS/CMC composite films, including four factors (CS, CMC, NaOH, urea) at four levels.

Level	Factor			
	A: CS % (*w*/*v*)	B: CMC %(*w*/*v*)	C: NaOH %(*w*/*v*)	D: Urea % (*w*/*v*)
1	1.0	1.0	1.0	4.0
2	1.5	1.5	1.5	8.0
3	2.0	2.0	2.0	12.0
4	2.5	2.5	2.5	16.0

**Table 7:** L_16_ (4^4^) orthogonal array design.

Run No.	A: CS %(*w*/*v*)	B: CMC %(*w*/*v*)	C: NaOH %(*w*/*v*)	D: Urea %(*w*/*v*)
A_1_	1.0	1.0	1.0	4.0
A_2_	1.0	1.5	1.5	8.0
A_3_	1.0	2.0	1.0	12.0
A_4_	1.0	2.5	2.5	16.0
A_5_	1.5	1.0	1.5	12.0
A_6_	1.5	1.5	1.0	16.0
A_7_	1.5	2.0	2.5	4.0
A_8_	1.5	2.5	2.0	8.0
A_9_	2.0	1.0	2.0	16.0
A_10_	2.0	1.5	2.5	12.0
A_11_	2.0	2.0	1.0	8.0
A_12_	2.0	2.5	1.5	4.0
A_13_	2.5	1.0	2.5	8.0
A_14_	2.5	1.5	2.0	4.0
A_15_	2.5	2.0	1.5	16.0
A_16_	2.5	2.5	1.0	12.0

## Data Availability

Available upon request.
